# Intracranial hemorrhage in COVID-19 patients during extracorporeal membrane oxygenation for acute respiratory failure: a nationwide register study report

**DOI:** 10.1186/s13054-022-03945-x

**Published:** 2022-03-28

**Authors:** Saskia von Stillfried, Roman David Bülow, Rainer Röhrig, Patrick Meybohm, Peter Boor, for the German Registry of COVID-19 Autopsies (DeRegCOVID), DeRegCOVID Collaborators#, Jana Böcker, Jens Schmidt, Pauline Tholen, Raphael Majeed, Jan Wienströer, Joachim Weis, Juliane Bremer, Ruth Knüchel, Anna Breitbach, Claudio Cacchi, Benita Freeborn, Sophie Wucherpfennig, Oliver Spring, Georg Braun, Christoph Römmele, Bruno Märkl, Rainer Claus, Christine Dhillon, Tina Schaller, Eva Sipos, Klaus Hirschbühl, Michael Wittmann, Elisabeth Kling, Thomas Kröncke, Frank L. Heppner, Jenny Meinhardt, Helena Radbruch, Simon Streit, David Horst, Sefer Elezkurtaj, Alexander Quaas, Heike Göbel, Torsten Hansen, Ulf Titze, Johann Lorenzen, Thomas Reuter, Jaroslaw Woloszyn, Gustavo Baretton, Julia Hilsenbeck, Matthias Meinhardt, Jessica Pablik, Linna Sommer, Olaf Holotiuk, Meike Meinel, Nina Mahlke, Irene Esposito, Graziano Crudele, Maximilian Seidl, Kerstin U. Amann, Roland Coras, Arndt Hartmann, Philip Eichhorn, Florian Haller, Fabienne Lange, Kurt Werner Schmid, Marc Ingenwerth, Josefine Rawitzer, Dirk Theegarten, Christoph G. Birngruber, Peter Wild, Elise Gradhand, Kevin Smith, Martin Werner, Oliver Schilling, Till Acker, Stefan Gattenlöhner, Christine Stadelmann, Imke Metz, Jonas Franz, Lidia Stork, Carolina Thomas, Sabrina Zechel, Philipp Ströbel, Claudia Wickenhauser, Christine Fathke, Anja Harder, Benjamin Ondruschka, Eric Dietz, Carolin Edler, Antonia Fitzek, Daniela Fröb, Axel Heinemann, Fabian Heinrich, Anke Klein, Inga Kniep, Larissa Lohner, Dustin Möbius, Klaus Püschel, Julia Schädler, Ann-Sophie Schröder, Jan-Peter Sperhake, Martin Aepfelbacher, Nicole Fischer, Marc Lütgehetmann, Susanne Pfefferle, Markus Glatzel, Susanne Krasemann, Jakob Matschke, Danny Jonigk, Christopher Werlein, Peter Schirmacher, Lisa Maria Domke, Laura Hartmann, Isabel Madeleine Klein, Constantin Schwab, Christoph Röcken, Johannes Friemann, Dorothea Langer, Wilfried Roth, Stephanie Strobl, Martina Rudelius, Konrad Friedrich Stock, Wilko Weichert, Claire Delbridge, Atsuko Kasajima, Peer-Hendrik Kuhn, Julia Slotta-Huspenina, Gregor Weirich, Peter Barth, Eva Wardelmann, Alexander Schnepper, Katja Evert, Andreas Büttner, Johannes Manhart, Stefan Nigbur, Iris Bittmann, Falko Fend, Hans Bösmüller, Massimo Granai, Karin Klingel, Verena Warm, Konrad Steinestel, Vincent Gottfried Umathum, Andreas Rosenwald, Florian Kurz, Niklas Vogt

**Affiliations:** 4 Center for Translational & Clinical Research (CTC-A), University Hospital RWTH Aachen, Aachen, Germany; 5 Institute for Medical Informatics, University Hospital RWTH Aachen, Aachen, Germany; 6 Institute of Neuropathology, University Hospital RWTH Aachen, Aachen, Germany; 7 Institute of Pathology, University Hospital RWTH Aachen, Aachen, Germany; 8 Anesthesiology and Intensive Care, University Hospital Augsburg, Augsburg, Germany; 9 Department of Gastroenterology, University Hospital Augsburg, Augsburg, Germany; 10 General Pathology and Molecular Diagnostics, University Hospital Augsburg, Augsburg, Germany; 11 Hematology and Oncology, University Hospital Augsburg, Augsburg, Germany; 12 Laboratory Medicine and Microbiology, University Hospital Augsburg, Augsburg, Germany; 13 Diagnostic and Interventional Radiology, University Hospital Augsburg, Augsburg, Germany; 14 Department of Neuropathology, Charité - Universitätsmedizin Berlin, corporate member of Freie Universität Berlin and Humboldt-Universität zu Berlin, Berlin, Germany; 15 German Center for Neurodegenerative Diseases (DZNE) Berlin, Berlin, Germany; 16 Cluster of Excellence, NeuroCure, Berlin, Germany; 17 Institute of Pathology, Charité - Universitätsmedizin Berlin, corporate member of Freie Universität Berlin and Humboldt-Universität zu Berlin, Berlin, Germany; 18 Department of Pathology, University Hospital Cologne, Cologne, Germany; 19 Institute of Pathology, University Hospital OWL of the Bielefeld University, Campus Lippe, Detmold, Germany; 20 Department of Pathology, Klinikum Dortmund, Dortmund, Germany; 21 Institute of Pathology, University Hospital Dresden, Dresden, Germany; 22 Gemeinschaftspraxis für Pathologie, Dresden, Germany; 23 Institute of Forensic Medicine, University Hospital Düsseldorf, Düsseldorf, Germany; 24 Institute of Pathology, University Hospital Düsseldorf, Düsseldorf, Germany; 25 Department of Nephropathology, University Hospital Erlangen-Nürnberg, Erlangen, Germany; 26 Institute of Neuropathology, University Hospital Erlangen-Nürnberg, Erlangen, Germany; 27 Institute of Pathology, University Hospital Erlangen-Nürnberg, Erlangen, Germany; 28 Institute of Pathology, University Hospital Essen, Essen, Germany; 29 Institute of Forensic Medicine, University Hospital Frankfurt, Frankfurt, Germany; 30 Senckenberg Institute of Pathology, University Hospital Frankfurt, Frankfurt, Germany; 31 Institute for Surgical Pathology, Medical Center-University of Freiburg, Freiburg, Germany; 32 Institute of Neuropathology, University Hospital Giessen and Marburg, Giessen, Germany; 33 Institute of Pathology, University Hospital Giessen and Marburg, Giessen, Germany; 34 Institute of Neuropathology, University Medical Center Göttingen, Göttingen, Germany; 35 Institute of Pathology, University of Göttingen, Göttingen, Germany; 36 Institute of Pathology, University Hospital Halle (Saale), Halle (Saale), Germany; 37 Institute of Legal Medicine, University Medical Center Hamburg-Eppendorf, Hamburg, Germany; 38 Institute of Medical Microbiology, Virology, and Hygiene, University Medical Center Hamburg-Eppendorf, Hamburg, Germany; 39 Institute of Neuropathology, University Medical Center Hamburg-Eppendorf, Hamburg, Germany; 40 Institute of Pathology, Hannover Medical School, Hannover, Germany; 41 Institute of Pathology, Heidelberg University Hospital, Heidelberg, Germany; 42 Department of Pathology, University Hospital Schleswig-Holstein, Kiel, Germany; 43 Department of Pathology, University Hospital Cologne, Lüdenscheid, Germany; 44 Institute of Pathology, Klinikum Magdeburg, Magdeburg, Germany; 45 Institute of Pathology, University Medical Center Mainz, Mainz, Germany; 46 Institute of Pathology, Ludwig-Maximilians-Universität Munich, Munich, Germany; 47 Department of Nephrology, TUM School of Medicine of Technical University of Munich, Munich, Germany; 48 Institute of Pathology, TUM School of Medicine of Technical University of Munich, Munich, Germany; 49 Gerhard Domagk Institute of Pathology, University Hospital Münster, Münster, Germany; 50 Institute of Pathology, University Hospital Regensburg, Regensburg, Germany; 51 Rostock University Medical Center, University Hospital Rostock, Rostock, Germany; 52 Institute of Pathology, Agaplesion Diakonieklinikum Rotenburg, Rotenburg, Germany; 53 Institute of Pathology and Neuropathology, University Hospital Tübingen, Tübingen, Germany; 54 Department of Pathology, Bundeswehrkrankenhaus Ulm, Ulm, Germany; 55 Institute of Pathology, University of Würzburg, Würzburg, Germany; 1 Institute of Pathology, University Hospital RWTH Aachen, Pauwelsstraße 30, 52074 Aachen, Germany; 2 Institute for Medical Informatics, University Hospital RWTH Aachen, Pauwelsstraße 30, 52074 Aachen, Germany; 3 Department of Anaesthesiology, Intensive Care, Emergency and Pain Medicine, University Hospital Würzburg, Oberdürrbacher Str. 6, 97080 Würzburg, Germany

**Keywords:** Autopsy, Registry, COVID-19, ECMO, Intracranial bleeding, Bleeding events

## Abstract

**Background:**

In severe cases, SARS-CoV-2 infection leads to acute respiratory distress syndrome (ARDS), often treated by extracorporeal membrane oxygenation (ECMO). During ECMO therapy, anticoagulation is crucial to prevent device-associated thrombosis and device failure, however, it is associated with bleeding complications. In COVID-19, additional pathologies, such as endotheliitis, may further increase the risk of bleeding complications. To assess the frequency of bleeding events, we analyzed data from the German COVID-19 autopsy registry (DeRegCOVID).

**Methods:**

The electronic registry uses a web-based electronic case report form. In November 2021, the registry included *N* = 1129 confirmed COVID-19 autopsy cases, with data on 63 ECMO autopsy cases and 1066 non-ECMO autopsy cases, contributed from 29 German sites.

**Findings:**

The registry data showed that ECMO was used in younger male patients and bleeding events occurred much more frequently in ECMO cases compared to non-ECMO cases (56% and 9%, respectively). Similarly, intracranial bleeding (ICB) was documented in 21% of ECMO cases and 3% of non-ECMO cases and was classified as the immediate or underlying cause of death in 78% of ECMO cases and 37% of non-ECMO cases. In ECMO cases, the three most common immediate causes of death were multi-organ failure, ARDS and ICB, and in non-ECMO cases ARDS, multi-organ failure and pulmonary bacterial ± fungal superinfection, ordered by descending frequency.

**Interpretation:**

Our study suggests the potential value of autopsies and a joint interdisciplinary multicenter (national) approach in addressing fatal complications in COVID-19.

**Supplementary Information:**

The online version contains supplementary material available at 10.1186/s13054-022-03945-x.

## Introduction

Venovenous extracorporeal membrane oxygenation (VV-ECMO) is used for refractory severe acute respiratory failure with a survival rate of about 50% [1]. The interface between blood and non-biological ECMO circuit elements requires therapeutic anticoagulation, predisposing patients to an increased risk of bleeding.

Based on a recent analysis from the Extracorporeal Life Support Organization (ELSO) registry including 7579 patients from 2007 to 2018, 37% experienced any bleeding event, and 21.2% experienced bleeding combined with a thrombotic event. While the most common bleeding events with cannulation (15.5%) and surgical site (9.6%) bleeding are easy to handle, intracranial hemorrhage occurred in only 4.5% and has been consistently associated with poor survival [2]. Other retrospective studies reported intracranial hemorrhage in 6–35.4% of patients during ECMO therapy [3–5].

Causes of intracranial hemorrhage in ECMO patients may be associated with heparin overdose, circuit-associated defibrination, thrombocytopenia, disseminated intravascular coagulation, acquired von Willebrand syndrome, and also COVID-19-associated endotheliitis. Importantly, intracranial hemorrhage was observed in many patients receiving VV-ECMO without coagulopathy or anticoagulant use [6].

In this respect, we analyzed preliminary data from the German COVID-19 Autopsy Registry [7], involving 63 deceased COVID-19 patients who received ECMO for acute respiratory failure. Specifically, we address the following questions, comparing COVID-19 autopsy cases that received ECMO support with cases that did not:

1. What differences in demographical characteristics exist?
2. What are the prevalences of bleeding events, specifically intracranial bleeding events found at autopsy?

Patient inclusion, data acquisition and management, and cohort stratification were performed as previously described [8] and are provided in the Additional file 1.

**Fig. 1:**
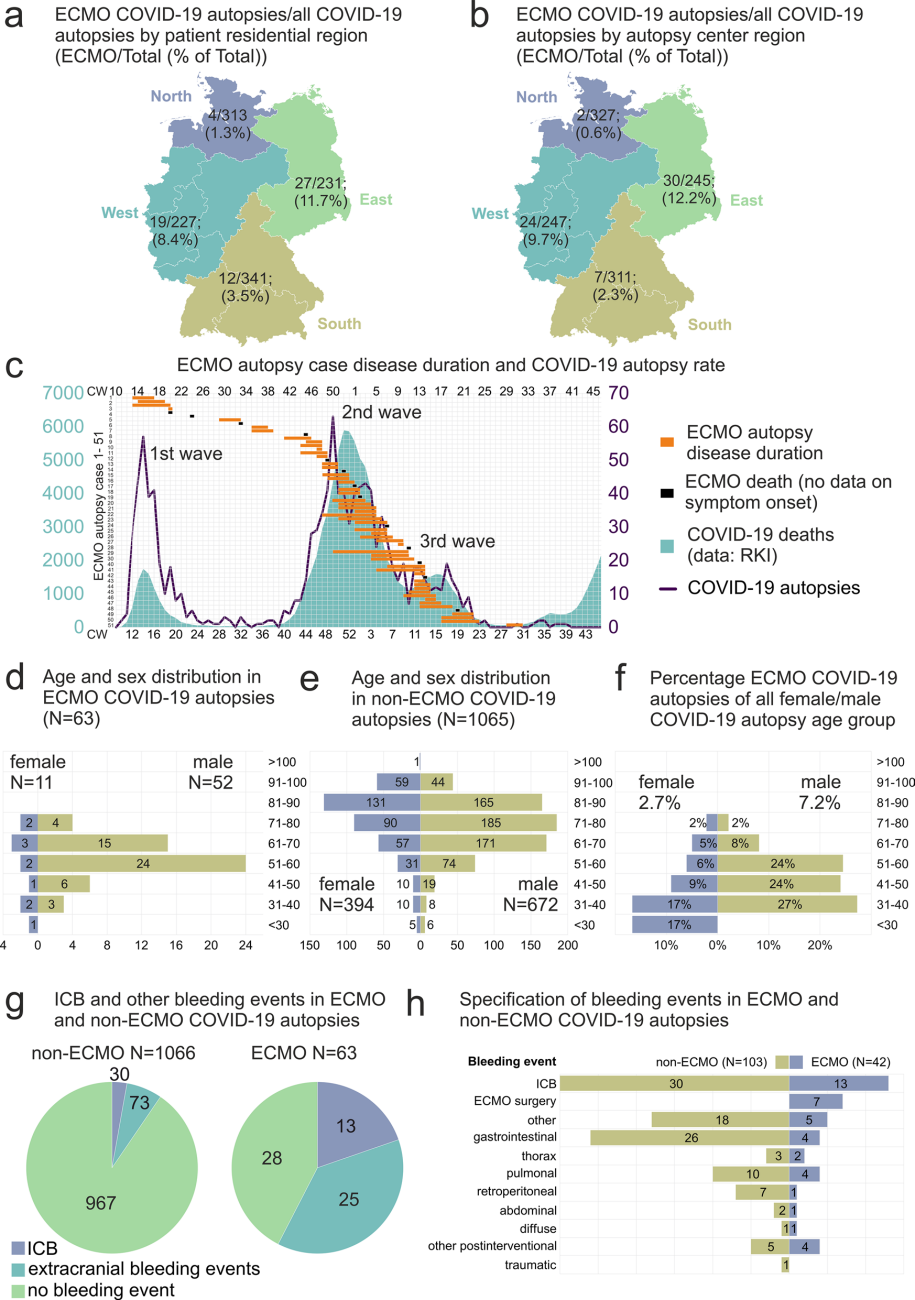
**a** Number of COVID-19 autopsy cases and percentage of COVID-19 autopsies after ECMO therapy by postal code of the deceased person (1 value missing of ECMO cases, 17 values missing of non-ECMO cases). **b** Number of COVID-19 autopsy cases and percentage of COVID-19 autopsies after ECMO therapy by postal code of the contributing center. **c** Individual disease duration (orange bars) or death date (black boxes, when no data on symptom onset/ first positive SARS-CoV-2 test was available) in *N* = 63 ECMO COVID-19 autopsy cases. **d** Age and sex distribution in COVID-19 autopsies after ECMO therapy (*N* = 63). **e** Age and sex distribution in COVID-19 autopsies without ECMO therapy (*N* = 1065, 1 value missing). **f** Age and sex distribution in COVID-19 autopsies as a percentage of respective age group. **g** Intracranial bleeding (ICB) and other hemorrhages in ECMO and non-ECMO COVID-19 cases. The associations between the variables ECMO and ICB and ECMO and any bleeding event were significant (both *p* value < 0.0001 Fisher’s exact test, two-tailed). Note that the number of bleeding events exceeds the number of patients, because in N = 3 non-ECMO, and *N* = 3 ECMO autopsies, both ICB and other bleeding events were present at the autopsy, respectively. **h** ECMO cases (violet) and non-ECMO cases (dark yellow) with any bleeding event. The number of extracranial bleeding events is higher compared to **h**, because, in *N* = 4 ECMO cases, two different extracranial bleeding events were documented. ICB, intracranial bleeding

For analyses, *N* = 1129 autopsy cases with positive COVID-19 test (preclinical, clinical, or post-mortem, point of care antigen test from nasopharyngeal or pulmonary swabs or PCR test from nasopharyngeal or pulmonary swabs or tissue samples) were eligible in the German COVID-19 Autopsy Registry (DeRegCOVID). 20–22% of the COVID-19 autopsy cases were located in the East and West, respectively and 28–30% in the North and South of Germany, respectively by patient residential region (Fig. 1a) or by the autopsy center region (Fig. 1b), respectively.

The percentage of COVID-19 ECMO autopsy cases of all autopsied cases increased with each pandemic wave, from 2% in the first pandemic wave to 6% in the second and 12% in the third wave (Fig. 1c).

*N* = 63 patients underwent ECMO therapy. The male to female ratio in this cohort was 5:1 with a homogeneous distribution of females over the different age range of < 30–80 years, and a male peak in the age range of 51–70 years (Fig. 1d). Of the remaining *N* = 1065 COVID-19 non-ECMO autopsy cases (one value missing), the male to female ratio was 2:1, with male peaks at 61–90 years and female predominance at > 90 years of age (Fig. 1e). The percentage of COVID-19 ECMO autopsy cases in the respective sex/age group was higher for males compared to females, however, in the younger age groups, total numbers were relatively low, resulting in large effects of single cases on the percentage (Fig. 1f).

Any kind of a bleeding event was found in 56% of ECMO cases (*N* = 35 cases) and 9% of non-ECMO cases (*N* = 100 cases, *p* value < 0.0001). Intracranial bleeding (ICB) was documented in *N* = 13 ECMO cases (21%) and in *N* = 30 non-ECMO cases (3%, *p* value < 0.0001, Fig. 1g). In ECMO patients with ICB, in three cases (*N* = 2 soft tissue bleeding due to cannulation and *N* = 1 epistaxis) and in non-ECMO patients with ICB, in three cases extracranial bleeding events were documented, respectively (*N* = 2 acute posthemorrhagic anemia not otherwise specified and *N* = 1 recurrent bleeding of the lower gastrointestinal tract, a detailed specification of bleeding events is provided in Fig. 1h). In 78% of ECMO cases and 37% of non-ECMO cases with ICB, the intracranial bleeding was classified as the immediate or underlying cause of death. The five most common immediate causes of death were multi-organ failure, DAD/ARDS, ICB, pulmonary bacterial ± fungal superinfection and extracranial bleeding events in *N* = 63 ECMO cases and DAD/ARDS, multi-organ failure, pulmonary bacterial ± fungal superinfection, pulmonary embolism, and ischemic heart disease in *N* = 1031 non-ECMO cases with the complete cause of death data (ordered by descending frequency).

VV-ECMO used in refractory severe acute respiratory failure is associated with an increased risk of bleeding, of which intracranial hemorrhage has been consistently associated with very poor survival. In this report, we analyzed data from the German Registry of COVID-19 Autopsies (DeRegCOVID) to gain further insights into COVID-19 ECMO autopsy cases with bleeding events in comparison to non-ECMO cases.

ECMO being more often documented in younger and male patients, is in line with data from the Extracorporeal Life Support Organization (ELSO) Registry [3].

The prevalence of any bleeding event in more than 50% of COVID-19 ECMO autopsy cases is higher compared to a previous multicenter observational study of 152 consecutive non-autopsy patients with severe COVID-19 supported by ECMO in four UK commissioned centers during the first wave of the COVID-19 pandemic (30.9% major bleedings) [9]. This might be explained by our cohort consisting of fatal cases, which may lead to an overrepresentation of cases with bleeding events. Also, all macroscopically identified bleeding events are documented, irrespective of major bleeding criteria [10], as these data are usually not available at autopsy. Our findings regarding the prevalence of intracranial bleeding and associated mortality are consistent with a report from three tertiary care ECMO centers in Germany and Switzerland [11]. In an observational study from Northern Germany, the observation of intracranial bleeding in COVID-19 non-autopsy ECMO patients (35.4%) was higher compared to our findings. In a study from a single tertiary center on 25 non-COVID ECMO autopsy cases, 52% had intracranial macrohemorrhages [12]. However, it is possible, that due to concerns regarding occupational hazards, the omission of brain examination especially during the first pandemic wave led to an underreporting of intracranial hemorrhage at autopsy in our cohort.

### Limitations

The registry only gathers data available to pathologists at the time of autopsy. The clinical information provided during autopsies is usually comprehensive, particularly regarding treatment approaches such as ECMO. Still, we cannot exclude missing data in the registry on potential ECMO therapy. Another limitation is the missing specific data on invasive ventilation therapy and missing reliable data on the mode and time of anticoagulant therapy in our cohort. Because we aimed at the broadest possible participation in the registry, the eCRF does not cover therapy and intensive care details and duration of ventilation or ECMO therapy. Considering that in more than 50% of our cohort, the immediate cause of death at autopsy was COVID-19 DAD/ARDS, it is likely that some of these patients underwent invasive ventilation therapy before death in hospitalized cases [8].

In conclusion, our report showed autopsy-confirmed increased prevalence of bleeding events and intracranial hemorrhages as causes of death in COVID-19 patients with ECMO treatment, compared to those without ECMO treatment. This illustrates the value of autopsies and a joint interdisciplinary multicenter (national) approach in addressing fatal complications in intensive care.

## Supplementary Information

**Additional file 1.** Supplementary Methods and Discussion.

## Data Availability

The datasets used and/or analyzed during the current study are available from the corresponding author on reasonable request.

## Abbreviations

ARDS: Acute respiratory distress syndrome
COVID-19: Coronavirus disease 2019
DAD: Diffuse alveolar damage
DeRegCOVID: German COVID-19 Autopsy Registry
eCRF: Electronic case report form
ELSO: Extracorporeal Life Support Organization
ICB: Intracranial bleeding
PCR: Polymerase chain reaction
RWTH Aachen University: Rhenish-Westphalian Technical University Aachen
SARS-CoV-2: Severe acute respiratory syndrome coronavirus 2
VV-ECMO: Venovenous extracorporeal membrane oxygenation
