# Supplementary material for: Intracranial hemorrhage in COVID-19 patients during extracorporeal membrane oxygenation for acute respiratory failure: a nationwide register study report
Source: Crit Care. 2022 Mar 28;26:83. doi: 10.1186/s13054-022-03945-x (PMC8958804; doi:10.1186/s13054-022-03945-x)
Supplement: Supplementary file 1 — Additional file 1. Supplementary Methods and Discussion. [file 13054_2022_3945_MOESM1_ESM.docx]

**Supplementary Methods**

Patient inclusion

The German Registry for COVID-19 autopsy registry (DeRegCOVID) is the electronic backbone of the project DEFEAT PANDEMIcs in the frame of the Network University Medicine, funded by the German Federal Ministry of Education and Research. It aims to gather data on (potentially all) COVID-19 autopsies in Germany, to support autopsy centers and researchers, provide central analyses, data curation and integration, particularly with other NUM projects. Participation is voluntary and biomaterials remain at the respective autopsy site (decentralized biobanking). Autopsy centers retain the rights to their autopsy data and biomaterials including publication. N=1229 autopsy cases have been registered between April 30^th^, 2020 until November 16^th^, 2021 in the DeRegCOVID. Of N=1229 registered autopsy cases, N=99 were excluded from all analyses in this study due to lacking information on a positive SARS-CoV-2 test result before or after death.

Data Acquisition

The data was recovered from the final autopsy report or the laboratory information system by a member of the autopsy team (physician or autopsy technician), scientific staff member or by a medical documentation assistant. All cases with a positive SARS-CoV-2 test (usually antigen tests from either nasopharyngeal or PCR from either nasopharyngeal swab or tissue), either preclinical, clinical or post-mortem, were been eligible for registration and analysis. In addition to full autopsy, minimal invasive autopsies were also eligible. In N=17 cases, biopsy was mentioned as sample type. Of note, brain examination at autopsy was not documented in N=19 ECMO patients (N=5 with ECMO and ICB) and in N=300 non-ECMO patients (N=5 non-ECMO with ICB) because of concerns about occupational exposure to infectious aerosols from sawdust when the skull was opened. This is especially true for the first wave of the pandemic, when autopsy rooms were not yet equipped with special bone saws with vacuum unit and saw dust filter.

Data Management

The acquisition of data is achieved with the Electronic Data System (EDC) LibreClinica (version 1.0.0rt snapshot). The information on intracranial and extracranial hemorrhage was retrieved from either clinical information or from the final autopsy report after the histological examination. The cause of death data was taken from the final autopsy report after the histological examination. The Nomenclature diagnosis was curated according to the ICD-11 International Classification of Diseases and Related Health Issues, Eleventh Revision, 1st ed. Cause of death data was centrally reviewed and curated following the ICD-10: International Statistical Classification of Diseases and Related Health Problems, Tenth Revision, 2nd ed., International Guidelines for Certification and Classification (Coding) of COVID-19 as Cause of Death, as previously reported [1]. Entries without cause of death sequences reported in combination with a nonspecific condition as the immediate cause of death or missing data in line 1a (e.g., cardiovascular failure without underlying disease) have been excluded (no ECMO cases, N=35 non-ECMO cases). The category “other” (Figure 1h) comprises N=1 epistaxis, N=1 diffuse bleeding from the oral cavity and N=18 unspecified hemorrhage or mention of anemia due to unspecified hemorrhage. The category “other postinterventional” (Figure 1h) comprises N=4 from punction or tracheostomy sites, N=1 hemorrhage after laceration of the femoral artery, N=1 hemorrhage after endoprosthetic surgery and N=1 patient with several hemorrhages (tracheal, femoral arterial and urethral). The intracranial bleeding (ICB) in N=13 ECMO cases was non-traumatic in all cases (N=1 subarachnoid, N=8 intracerebral, N=4 intracranial not otherwise specified and traumatic in N=2 of N=30 non-ECMO cases.

Coordinated stratification by the region

The first digit of the German zip code at the deceased persons home address was used to stratify the cohort by the region, as described previously [1]. Shortly, zip codes start with the digits 0-1 being assigned to the "East", digits 2 to the "North", digits 3-6 to the "West", and digits 7-9 to "The South". It should be noted that stratification by postcode results in different numbers compared to adding the cases contributed of regional centers. This may be due to patients being transferred from one region to another due to ICU and specialized ECMO capacity, but also due to residents living or being hospitalized in a region that is not identical to the region in which they are registered officially.

Cohort stratification by pandemic wave

Pandemic waves were defined based on COVID-19 death data from the Robert Koch Institute as follows: 1st wave, from calendar week 10 to 31, inclusive, in 2020. Calendar weeks 32 in 2020 to 11, inclusive, in 2021 were selected for the 2nd wave [2]. The 3rd wave was defined as beginning calendar week 12 to 32, inclusive, in 2021.

Statistics

The association between two variables was tested with Fisher’s exact test, two tailed, using https://www.graphpad.com/quickcalcs/contingency2/.

**Supplementary Discussion**

The autopsied ECMO patients are distributed heterogeneously over four German regions. The lower percentage of COVID-19 ECMO autopsy cases in the North can be explained by the fact that in Hamburg, in a collaboration between forensic medicine and the local health authority, all COVID-19 positive deceased persons were autopsied during the first pandemic wave (Q1 and Q2 in 2020), leading to a higher percentage of non-hospitalized deceased COVID-19 patients that underwent autopsy [3].

Other limitations include that the autopsy registry does not yet include all COVID autopsies in Germany. We estimate that about 10% of autopsied cases in Germany are missing for complete coverage. Still, we assume the collective is well representative [1]. Other limitations include the retrospective nature of the registry data. Also, post-mortem imaging before autopsy was performed only by single centers in Germany due to capacity issues.

**Supplementary References**

1. von Stillfried S, Bulow RD, Röhrig R, Boor P: **First report from the German COVID-19 autopsy registry**. *The Lancet Regional Health - Europe* 2022, in press.

2. **[Deaths by date of death] German.** [<https://www.rki.de/DE/Content/InfAZ/N/Neuartiges_Coronavirus/Projekte_RKI/COVID-19_Todesfaelle.html>]

3. Fitzek A, Schadler J, Dietz E, Ron A, Gerling M, Kammal AL, Lohner L, Falck C, Mobius D, Goebels H *et al*: **Prospective postmortem evaluation of 735 consecutive SARS-CoV-2-associated death cases**. *Sci Rep* 2021, **11**(1):19342.
